# A Survey of Dog Owners in Remote Northern Australian Indigenous Communities to Inform Rabies Incursion Planning

**DOI:** 10.1371/journal.pntd.0004649

**Published:** 2016-04-26

**Authors:** Emily G. Hudson, Navneet Dhand, Salome Dürr, Michael P. Ward

**Affiliations:** 1 Faculty of Veterinary Science, The University of Sydney, Camden, New South Wales, Australia; 2 Veterinary Public Health Institute, University of Bern, Bern, Switzerland; American Heart Association, UNITED STATES

## Abstract

Australia is underprepared for a rabies incursion due to a lack of information about how a rabies outbreak would spread within the susceptible canine populations and which control strategies would be best to control it. The aim of this study was to collect information to parameterize a recently developed dog rabies spread model as well as use this information to gauge how the community would accept potential control strategies. Such information–together with model outputs–would be used to inform decision makers on the best control strategies and improve Australia’s preparedness against a canine rabies incursion. The parameters this study focussed on were detection time, vaccination rates and dog-culling and dog movement restriction compliance. A cross-sectional survey of 31 dog-owners, using a questionnaire, was undertaken in the five communities of the Northern Peninsular Area (NPA) in northern Australia regarding community dog movements, veterinary visits, reporting systems, perceptions of sick dogs and potential human behaviours during hypothetical rabies outbreaks. It highlighted the significant shortfalls in veterinary care that would need to be vastly improved during an outbreak, who educational programs should be targeted towards and which dog movements should be restricted. The results indicate that men were significantly more likely than women to allow their dogs to roam and to move their dogs. The current low vaccination rate of 12% highlighted the limited veterinary services that would need to be substantially increased to achieve effective rabies control. Participation in mass vaccination was accepted by 100% of the respondents. There was lower acceptance for other possible rabies control strategies with 10–20% of the respondents stating a resistance to both a mass culling program and a ban on dog movements. Consequently, movement bans and mass dog culling would have limited effectiveness as a control strategy in the NPA community. More than half of the respondents said that they would report their sick dogs within a week. This would lead to a much more optimistic rabies detection time than observed in other regions with recent dog rabies outbreaks. Findings from this study can be used to parameterize a recently developed dog rabies spread model as well as to develop informed policies for managing a future rabies incursion, thus improving Australia’s preparedness against a canine rabies incursion.

## Author Summary

Australia is underprepared for a rabies incursion due to limited information about how a rabies outbreak would behave and which control strategies would be best to control it. A disease spread model of rabies has been developed to help policy-makers decide on the best response to a rabies incursion. However, data to inform this model are lacking. Therefore, the aim of this study was to gather information to parameterize the existing rabies spread model and to gauge how the community would accept potential control strategies. A survey of dog-owners, using a questionnaire, was undertaken in five remote, indigenous communities in northern Australia. We found that compared to women, men were more likely to allow their dogs to roam and to move their dogs. The current vaccination rates in these dog populations are low due to limited veterinary services. This would make delivery of vaccine in the event of a rabies incursion potentially challenging. However, compliance of dog owners with mass vaccination campaigns would be high. However, compliance with movement control of dogs might be problematic, as would the mass culling of dogs, although, rabies detection following an incursion could optimistically occur within a week.

## Introduction

**Fig 1:**
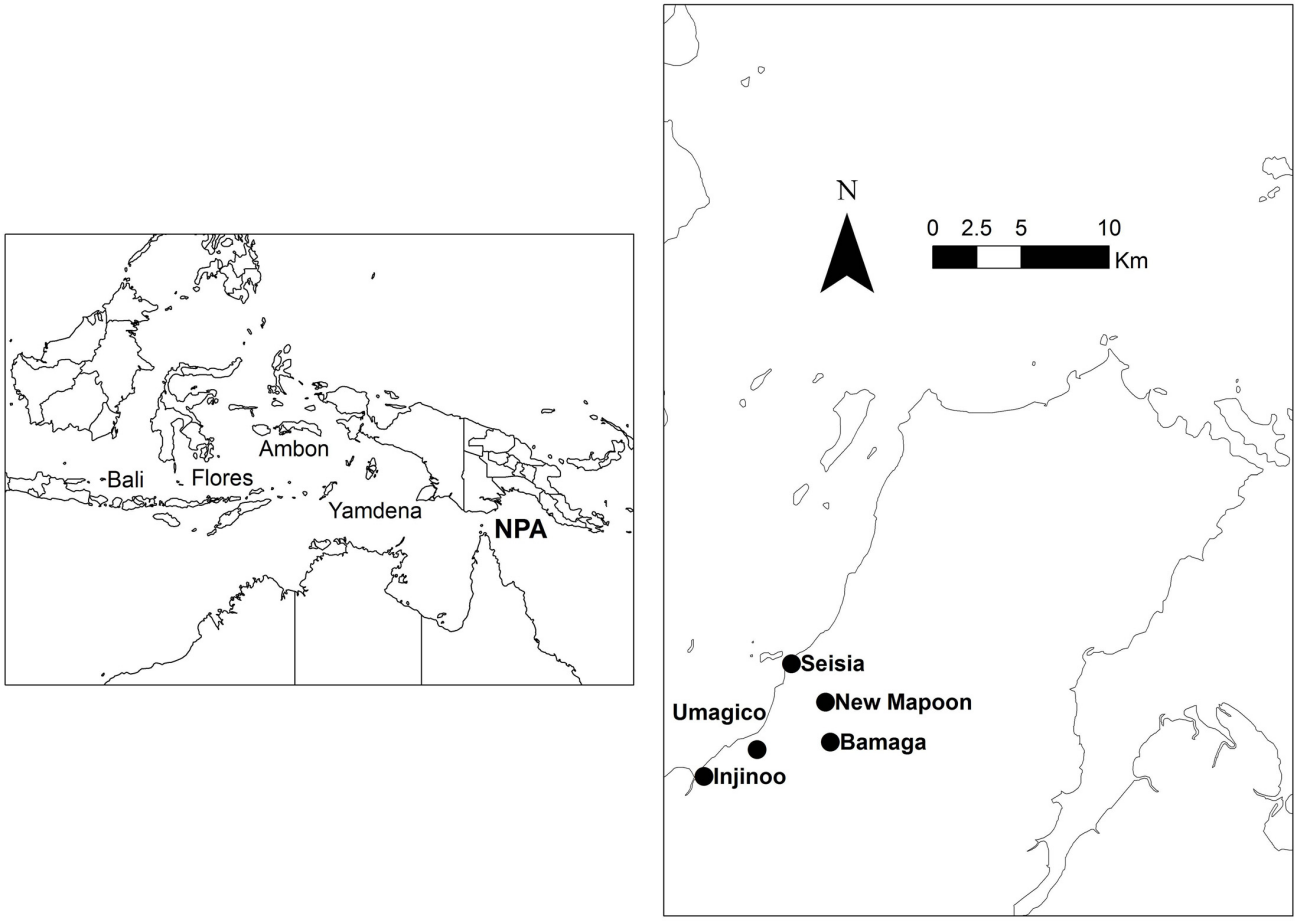
Map of the study sites where a questionnaires survey of dog owners about rabies control strategies was conducted. Left: The Northern Peninsular Area (NPA) in relation to the rest of Australia. Right: Arrangement of communities within the NPA.

Rabies is an acute viral zoonosis that causes approximately 60,000 human deaths annually, despite being preventable [1]. The disease occurs worldwide, with half of the annual deaths occurring in Asia [1, 2]. Although Australia is one of the few countries free of canine rabies [3], the increasing number of islands becoming infected in Indonesia − including Bali, Flores, Ambon and Yamdena − has brought rabies within 300km of Australia (Fig 1) and the risk of an incursion is escalating [4–6].

The Northern Peninsula Area (NPA) − located in far north Queensland and adjacent to the Torres Strait − is the most likely location for a rabies incursion [7, 9]. Not only is it close to Indonesia, the NPA has similar characteristics to Bali, which experienced an incursion in 2008 [4, 7]. These include large populations of free roaming domestic dogs that can facilitate disease spread, inefficient surveillance and vaccination, plus limited access for dog owners to health resources [6, 8]. It is vital to understand how a rabies outbreak would spread in such high-risk areas of northern Australia in order to develop response plans [9, 10]. It is also necessary to understand community attitudes and perceptions towards control strategies to anticipate potential barriers to implementation. However, such information is lacking because − except for one isolated incursion in the 1860s − there has never been an outbreak of rabies in Australia. Consequently, Australia is underprepared for a potential rabies incursion [7].

Epidemiological models are powerful tools that provide insight on disease spread and impacts [11–15]. Rabies epidemiological models have mainly been used in rabies endemic regions to refine and evaluate control strategies. Such models are also invaluable in rabies free areas to simulate outbreaks and evaluate potential control strategies. However, models for use in rabies free regions are scarce. There is only one recently developed model able to simulate a rabies outbreak in Australia and how different control strategies would influence its spread [9]. This model was based on limited data and many assumptions from northern Australia (the NPA and East Arnhem shire) and as a result, some key parameters for detection time and the main control strategies (rates for dog vaccination, dog culling and movement restrictions of dogs) are not based on extensive empirical data [9]. This study aimed to provide these data and to enable more accurate parameterization of the model to increase its predictive power. In addition, the study assessed community attitudes towards potential control strategies, and thus their efficacy in the event of a rabies incursion into the NPA.

## Methods

### Survey Area

A household survey was conducted in the NPA, which is a local government area located at the northern tip of Cape York (Fig 1). The NPA has a land area of 105,691 ha and consists of five communities: Bamaga, Umagico, Injinoo, New Mapoon and Seisia with the distances between individual communities ranging from 2 to 5 km. In 2011 there were 811 households in the NPA and a population of 2,298 [16]. A census of dogs in the NPA, conducted in 2009, estimated the total dog population to be 437 within 276 households, with 1.6 dogs per household [17].

### Questionnaire Design

A questionnaire was designed (S1 Questionnaire) with a combination of closed- and open-ended questions. The majority were closed questions with yes/no options. Some hypothetical questions were included to gauge what respondents would do if there were a rabies outbreak in community dogs. The questionnaire had four sections: 1. human and dog demographics; 2. dog movements within the community; 3. current dog vaccination rates; and 4. estimated detection time for rabies. Section 1 sought information on human demographics and the age, sex and number of dogs owned in that household. In Section 2, questions were asked about dog movements, either free roaming movements or human mediated movements, and whether the respondents ever restricted their dogs in the preceding 12 months. Some questions also focussed on whether the owners would hypothetically change their dog restriction practices if there were a rabies outbreak. In Section 3, the current level of vaccination (such as for canine parvovirus, distemper and infectious hepatitis) in the dog population was estimated based on when the dogs were last taken to a veterinarian, when their last vaccination was and what it was given for. Respondents were also asked whether they would hypothetically vaccinate their dogs if a rabies outbreak occurred in their community as well as if they would euthanize their dogs during an outbreak. Section 4 focused on estimating a potential detection time of a disease outbreak by asking questions about how respondents would describe sick dogs, how long they would wait until seeking advice about their sick dog and where they would go for veterinary services if they had been bitten by a dog in the last 12 months, if their dog had been bitten by another dog in the last 12 months and if they had reported any of these incidents.

### Survey Procedure

The survey was conducted from June 15 to 18, 2015. The questionnaire was administered with the assistance of the local NPA Animal Management Worker (AMW), who identified eligible and willing participants, explained the reasons for the survey and provided information regarding confidentiality and confirmed verbal consent to participate prior to the interview. The AMW is a community member and local government employee who has undergone basic training in animal management and handling and is usually the first point of contact for community members for animal related problems. They are also responsible for distribution of animal related information within the community. All questionnaires were administered in person as face-to-face interviews by the first author. Only community residents who were dog owners were selected. For ethical reasons, an age restriction of 18 years or older was also applied for the respondent selection. All questions were asked and answers were given in English, with interpretation help from the AMW as needed. Questionnaires were generally conducted at the dog owner’s place of residence, with their dogs usually being present. A small number of interviews were conducted at the respondents’ workplace. The survey was approved by The University of Sydney’s Human Research Ethics Committee (number 2013/757).

### Association Analysis

The data were compiled in Microsoft Excel (S1 Dataset). Responses to open-ended questions were categorised to allow for easier interpretation and analysis. For example, the answers for how respondents would describe a sick dog were condensed into categories such as behaviour, skin condition, physical ailments and body condition. Descriptive analyses were carried out for each question and included count and percentage for categorical responses (e.g. “Did you move your dog(s) in the previous 12 months?”), and median and range for continuous responses (e.g. “How many dogs did you own in the previous 12 months?”). The demographic variables were condensed into two variables per category for statistical analysis: male and female for human gender, young (20–39 years) and old (>-40 years) for human age; and ≥3 versus ≤2 dogs per house for number of dogs per house. Fisher’s exact test was used to explore the associations and due to the relatively small sample size, a liberal significance level of P = 0.1 was used to identify significant associations. The analyses were carried out using the R statistical program (Version 0.98.1091) [18].

## Results

### Human and Dog Demographics

Thirty-one dog owners were interviewed in the survey with each person representing one household. Based on the 2009 survey, which estimated 276 dog-owning households, this represents approximately 11% of the dog owning households in the NPA [17]. All five communities were represented in the survey. Umagico and Seisia were both overrepresented: the percentage of dog owning houses surveyed (14% and 29%, respectively) was greater than the overall survey percentage. Bamaga and New Mapoon were under represented (each approximately 7%) and Injinoo was substantially underrepresented (3%). The age of most respondents was between 40−49 (42%) years and most (81%) were male. This differs from the overall population in 2011, when the median age was 22 years and there was nearly a 1:1 male to female ratio (Table 1) [16]. The difference in population ages between the study and the census data was a result of the ethics restrictions allowing for only owners over the age of 18. This skewed the study age towards the older end of the scale compared to the census data that includes all ages.

**Table 1:** Demographics of 31 surveyed dog-owner respondents in the Northern Peninsular Area (NPA), Australia in 2015 compared to 2011 census data.

Variable	Category	(No.) %	ABS census data (%)
Community of residence	Bamaga	26 (8)	45.6
	Seisia	32 (10)	8.8
	Injinoo	3 (1)	20.7
	New Mapoon	13 (4)	12.6
	Umagico	26 (8)	12.2
Human gender	Female	19 (6)	51.5
	Male	81 (25)	48.5
Human age	20–29	10 (3)	15.9
	30–39	19 (6)	13.0
	40–49	42 (13)	10.7
	50–59	23 (7)	9.0
	60+	7 (2)	6.6
Employment	Casual	10 (3)	Not reported
	Part	3 (1)	20.9
	Full	77 (24)	64.5
	Retired	3 (1)	Not reported
	Unemployed	3 (1)	8.6
	No answer	3 (1)	
Education^1^	TAFE^2^	3 (1)	Not reported
	Year 10	29 (9)	Not reported
	Year 11	16 (5)	Not reported
	Year 12	36 (11)	Not reported
	University	3 (1)	Not reported
	No answer	13 (4)	
Ethnicity	Aboriginal	13 (4)	27.6
	Torres Strait Islander	52 (16)	51.4
	Both^3^	19 (6)	Not reported
	Non indigenous	16 (5)	7.9

^1^ Highest education received by respondents asked in this study.

^2^ Technical and Further Education

^3^The ABS does not report those who identified as both Aboriginal and Torres Strait Islander. These people had to choose one or the other.

**Fig 2:**
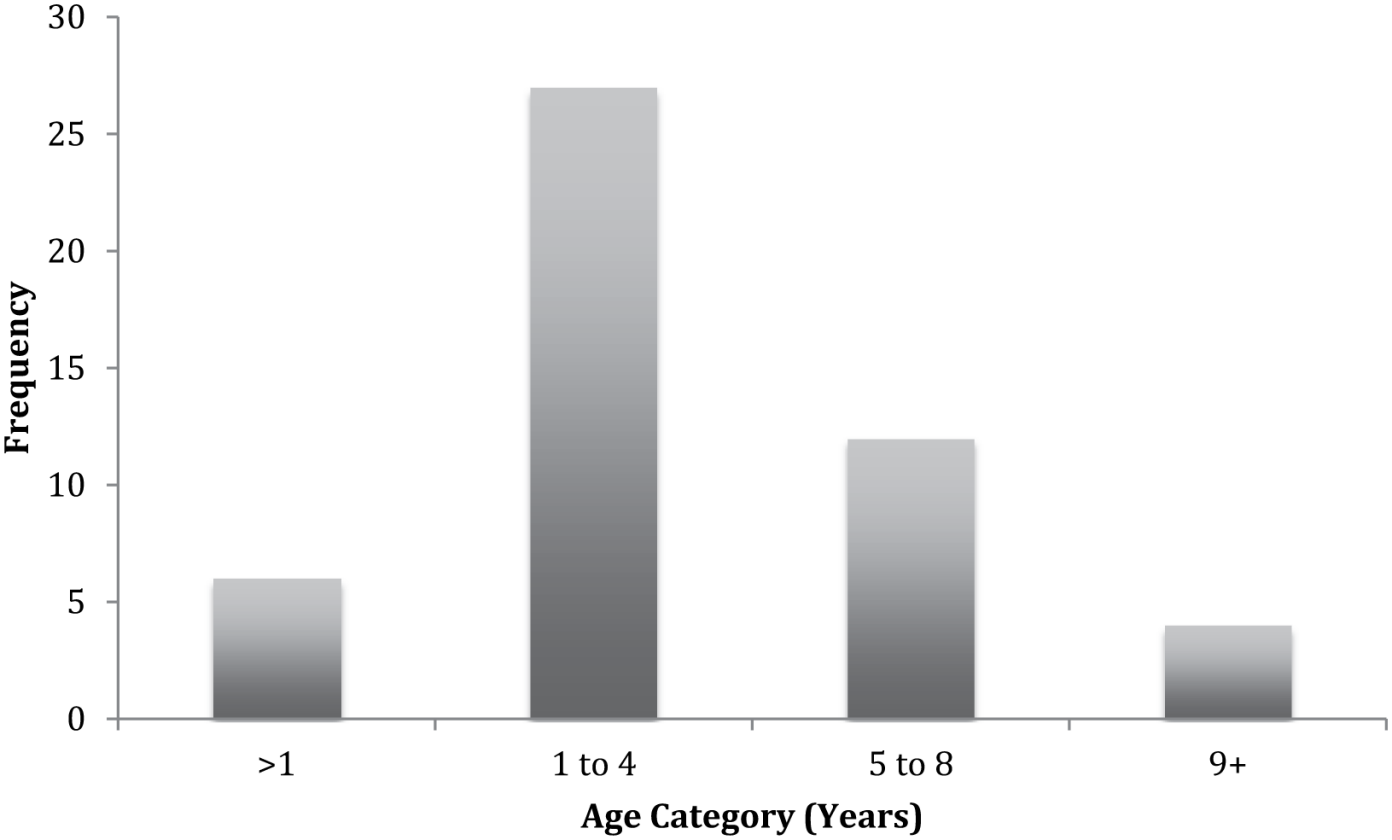
Frequency distribution of dog ages in communities of the Northern Peninsular Area (NPA), Australia. Most dogs were between 1 and 4 years of age. The maximum age was 10 years old. Twenty-five dogs were of unknown age. The 31 participants owned a total of 74 dogs (average 2.39 dogs per household). The range of dogs owned was 1–5 dogs per house.

A total of 74 dogs were owned by the 31 respondents, an average of 2.4 dogs per household (range 1−5). Most dogs in these households were 1−4 years of age (Table 2). However, a large proportion (34%) of dogs were of unknown age (Fig 2). The dog sex ratio was equally distributed (51% males, 49% females) and a small proportion were de-sexed (18%).

**Table 2:** Demographic data for the 74 dogs owned by the 31 respondents in a survey conducted in the indigenous communities of the Northern Peninsular Area (NPA), Australia, 2015.

Variable	Category	Number	%
Dog gender	Female	36	49
	Male	38	51
Desexed	Yes	13	18
	No	61	82
Dog age (years)	<1	6	8
	1 to 4	28	38
	5 to 8	11	15
	9+	4	5
	Unknown	25	34
Dog birth place	Local^1^	41	55
	Weipa	5	7
	Cairns	13	18
	Other^2^	7	10
	Unknown	8	11

^1^ Local = within Northern Peninsula Area Communities

^2^ Other includes Lockhart, Archer River, Torres Strait Islands, Townsville and Thursday Island (all locations are in North Queensland).

### Dog Movements within the Communities

More than half of the interviewed community members (58%) allowed their dogs to roam within their community of residence and many dogs had been moved by owners during the preceding 12 months, mostly for pig hunting and camping (Table 3). Most respondents had imposed some sort of restriction on the movement of their dogs in the last 12 months, by closing the gate, chaining the dogs or keeping them inside the house. In the event of a disease outbreak the proportion of respondents who would restrict their dogs' movements increased by 6% from 87% to 93% (Table 3). Of the respondents that would restrict their dogs during a disease outbreak, most would impose more restrictive conditions than in normal circumstances. For instance, if the gate was closed in normal circumstances, the owner would chain or keep the dog inside during a disease outbreak. Likewise, if the dog were chained normally, during an outbreak the owner would keep the dog inside.

**Table 3:** Dog movements between and within communities of the Northern Peninsular Area (NPA), Australia as reported by dog owners in a survey conducted in 2015.

Variable	Category	Number	%
Dog roaming	Yes	18	58
	No	13	42
Human mediated movement	Yes	16	52
	No	15	48
Where moved to	Camping	4	25
	Hunting	11	69
	Beach	1	6
Restriction	Yes	27	87
	No	4	13
Disease restriction	Yes	29	93
	No	2	7
Increased restrictions^1^	Yes	23	79
	No	6	21

^1^ Of the respondents that said “Yes” to restricting their dog, which respondents would impose harsher restrictions than normal i.e. from closed gate in normal situations to chaining or keeping dog(s) inside during a disease outbreak

### Current Dog Vaccination Rates

The minority of respondents visited a veterinarian in the preceding 12 months; most had visited a veterinarian in 2013 or earlier (Table 4). More than half of the respondents said their dogs have had a “needle” in their lifetime, with 40% of these respondents reporting them as vaccinations. As the term “needle”was substituted in the questionnaire for any injections given to ease communication, the rest of the needle administrations (60%) were for reasons such as mange and worming treatments and arthritis alleviation. However, of needles given, only a small proportion had been during the preceding 12 months and not necessarily administered by a veterinarian. All respondents were willing to vaccinate their dogs in the event of a disease outbreak. Only a small number of respondents (10%) were opposed to or unsure of euthanizing their dogs during a disease outbreak if they were prompted by the AMW or by local influences. However, the number of respondents opposed to euthanasia increased by 6% if they were forcibly told to euthanize their dogs by a non-local government official.

**Table 4:** Dog health treatment details for the 74 dogs surveyed in the Northern Peninsular Area (NPA), Australia, 2015.

Variable	Category	Number	%
Last veterinary visit	2015	1	3
	2014	7	23
	2013 and earlier	15	48
	Never	7	23
	Unknown	1	3
Needle in lifetime	Yes	21	68
	No	9	29
	Unknown	1	3
Latest needle^1^	2015	3	14
	2014	4	19
	2013 and earlier	13	62
	Unknown	1	5
Reason for latest needle^2^	Worming/mange	16	57
	Vaccination	11	39
	Other^3^	1	4
Vaccination if disease	Yes	31	100
	No	0	0
Vaccination if told to	Yes	31	100
	No	0	0
Euthanize if disease	Yes	28	90
	No	2	7
	Unsure	1	3
Euthanize if told to	Yes	26	84
	No	4	13
	Unsure	1	3

^1^ Percentages of the 21 respondents that replied “Yes” to if their dog had a needle in their lifetime.

^2^ Some dogs had more than one needle so the percentage is derived from the total number of needles given, which were 28.

^3^ Other reasons for needle were arthritis treatments

### Estimated Detection Time

The most common signs respondents used to determine if a dog was sick were physical ailments (scratches, limping, pus) and skin conditions (mange and hair loss). Other signs identified were body condition (skinny), behavioural changes (not coming when called or very lethargic) and gastrointestinal signs (diarrhoea and vomiting) (Table 5). Most respondents would wait a few days before reporting their sick dog and the majority would report it to the animal management worker. A small number of respondents would report a sick dog to the veterinarian (5), ranger (1), hospital (1) and manager of the local abattoir (1). Half (50%) of the respondents who were bitten by dogs reported their injury and the event. Conversely, only 10% of respondents reported that their dogs had been bitten by other dogs.

**Table 5:** Dog owners' responses to sick dogs in the Northern Peninsular Area (NPA), Australia that encompasses identification of sick dogs, reporting systems, veterinary services, human-dog bites and dog-dog bites from a survey of dog-owners conducted in the NPA in 2015.

Variable	Category	Number	%
Description of sick dog^1^	Skin conditions	20	39
	Physical ailments	14	61
	Body condition	11	43
	Gastrointestinal signs	4	29
	Behaviour change	13	7
	Unknown	2	21
Correct identification of 'sick' dog	Picture A	27	58
	Picture B	30	42
	Picture C	31	36
	Picture D	31	36
	Picture E	30	12
Sick in last 12 months	Yes	12	16
	No	19	74
Signs of sick dogs^2^	Skin conditions	6	14
	Physical ailments	4	9
	Gastrointestinal signs	1	3
	Behaviour	3	39
Reported sick dog	Yes	7	7
	No	5	45
How long until report a sick dog	Immediately	11	9
	Within a week	11	7
	More than a week	4	93
	Unknown	5	50
Who would they report to^3^	AMW^4^	26	50
	Vet	5	61
	Other^5^	3	39
	No one	1	10
Veterinary services	AMW^4^	12	90
	Abattoir manager	2	39
	Outside NPA^6^	14	61
	Unknown	3	43
Human bites	Yes	2	29
	No	29	7
Human bites reported	Yes	1	21
	No	1	58
Dog bites	Yes	19	42
	No	12	36
Dog bites reported	Yes	2	36
	No	17	12

^1^ Many respondents replied with multiple signs so percentages derived from a total of 64 responses

^2^ Only respondents that said their dogs were sick in the last 12 months replied to this and many saw more than one sign so percentage based on a total of 14 signs

^3^ Some respondents may report a sick dog to more than one person so percentages based on a total of 35 answers

^4^ AMW = animal management worker

^5^ Other includes rangers, hospital and local abattoir manager

^6^ Outside the NPA includes Thursday Island, Cairns and Weipa

### Association Analysis

There were few significant associations between human demographic and behavioural and knowledge variables (Tables 6 and 7). Men were 9.8 times more likely to allow their dogs to roam than women and only men took their dogs outside of the community, mainly for pig hunting (P = 0.007 and P = 0.059 respectively; Table 6). Having two or less dogs in a house meant there was significantly lower chance of one of the dogs being sick in the last 12 months compared to houses with three or more dogs (P = 0.008; Table 7), whilst younger dog owners were 5.01 times more likely to have had more dogs sick in the last 12 months than older dog owners (P = 0.058; Table 6). Although not statistically significant, there was a trend between having >2 dogs in the house and having more dog-dog bites (P = 0.15; Table 7) as well as more human mediated movements (P = 0.15; Table 7).

**Table 6:** Association analysis using Fisher’s exact test between human demographics data (gender and age) and selected variables from a survey of dog-owners conducted in the Northern Peninsula Area (NAP), Australia in 2015. A liberal significance level of 0.1 was used to determine significant associations.

	Human Gender					Human Age				
	M	F	Total	OR	P- Value	Young	Old	Total	OR	P-value
Dog roaming										
Yes	17	1	18			5	13	18		
No	8	5	13			4	9	13		
Total	25	6	31	9.8	0.059^2^	9	22	31	0.87	1
Human mediated movement										
Yes	16	0	16			3	9	12		
No	9	6	15			6	13	19		
Total	25	6	31	N/A^3^	0.007^2^	9	22	31	0.73	1
Movement restriction										
Yes	21	6	27			9	18	27		
No	4	0	4			0	4	4		
Total	25	6	31	0	0.56	9	22	31	N/A	0.3
Disease restriction										
Yes	23	6	29			9	20	29		
No	2	0	2			0	2	2		
Total	25	6	31	0	1	9	22	31	N/A	1
Veterinary visit										
Yes	17	6	24			7	16	24		
No	7	0	7			2	5	7		
Total	24	6	30^1^	0	0.29	9	21	30^1^	1.09	1
Needle in life										
Yes	16	5	21			5	16	21		
No	8	1	9			4	5	9		
Total	24	6	30^1^	0.41	0.64	9	21	30^1^	0.4	0.39
Picture A										
Yes	21	6	27			9	18	27		
No	4	0	4			0	4	4		
Total	25	6	31	0	0.56	9	22	31	N/A	0.3
Picture B										
Yes	24	6	30			9	21	30		
No	1	0	1			0	1	1		
Total	25	6	31	0	1	9	22	31	N/A	1
Picture E										
Yes	1	0	30			0	1	1		
No	24	6	1			9	21	30		
Total	25	6	31	N/A	1	9	22	31	0	1
Sick in last 12 months										
Yes	9	3	12			6	6	12		
No	16	3	19			3	16	19		
Total	25	6	31	0.57	0.65	9	22	31	5.01	0.06^2^
How long until report										
Within a week	19	3	22			5	17	22		
More than a week	2	2	4			2	2	4		
Total	21	5	26^1^	5.72	0.15	7	19	26^1^	3.11	0.29
Bitten by dog										
Yes	2	0	2			0	2	2		
No	23	6	29			9	20	29		
Total	25	6	31	N/A	1	9	22	31	0	1
Dog bitten by dog										
Yes	17	2	19			5	14	19		
No	8	4	12			4	8	12		
Total	25	6	31	4.04	0.17	9	22	31	0.72	0.7
Number of dogs above median (2)										
Yes	11	2	19			4	9	13		
No	14	4	22			5	12	17		
Total	25	6	31	1.54	1	9	22	31	1.06	1

^1^ Some respondents replied with “I’m not sure” which was excluded, leading to the variation in totals.

^2^ Indicates association was considered significant

^3^ N/A = odds ratio was calculated as infinity

**Table 7:** Association analysis using Fisher’s exact test between human demographics data (ethnicity and number of dogs owned) and selected variables from a questionnaire survey of dog owners conducted in the Northern Peninsula Area (NPA), Australia in 2015. A liberal significance level of 0.1 was used to determine significant associations.

	Ethnicity					Number of Dogs				
	Torres Strait Islander	Other	Total	OR	P-value	≤2	≥3	Total	OR	P-value
Dog roaming										
Yes	6	3	9			10	8	18		
No	10	12	22			8	5	13		
Total	16	15	31	2.33	0.43	18	13	31	0.79	1
Human movement										
Yes	7	9	16			7	9	16		
No	9	6	15			11	4	15		
Total	16	15	31	0.53	0.48	18	13	31	0.3	0.15
Restriction										
Yes	14	13	27			15	12	27		
No	2	2	4			3	1	4		
Total	16	15	31	1.07	1	18	13	31	0.43	0.62
Disease restriction										
Yes	14	15	29			16	13	29		
No	2	0	2			2	0	2		
Total	16	15	31	0	0.48	18	13	31	0	0.5
Veterinary visit										
Yes	12	11	23			12	11	23		
No	4	3	7			6	1	7		
Total	16	14	30^1^	0.82	1	18	12	30^1^	0.19	0.19
Needle in life										
Yes	10	11	21			10	11	21		
No	6	3	9			7	2	9		
Total	16	14	30^1^	0.47	0.44	17	13	30^1^	0.27	0.23
Picture A										
Yes	13	14	27			17	10	27		
No	3	1	4			1	3	4		
Total	16	15	31	0.32	0.6	18	13	31	4.83	0.28
Picture B										
Yes	16	14	30			17	13	30		
No	0	1	1			1	0	1		
Total	16	15	31	N/A^3^	0.48	18	13	31	0	1
Picture E										
Yes	0	1	1			1	0	1		
No	16	14	30			17	13	30		
Total	1	15	31	0	0.48	18	13	31	N/A^3^	1
Sick in last 12 months										
Yes	8	4	12			3	9	12		
No	8	11	19			15	4	19		
Total	16	15	31	2.66	0.27	18	13	31	0.1	0.008^2^
How long until report										
Within a week	10	14	24			12	10	22		
More than a week	3	1	4			3	1	4		
Total	13	13	26^1^	0.25	0.31	15	11	26^1^	0.41	0.61
Bitten by dog										
Yes	0	2	29			1	1	2		
No	16	13	2			17	12	29		
Total	16	15	31	0	0.23	18	13	31	0.77	1
Dog bitten by Dog										
Yes	12	7	19			9	10	19		
No	4	8	12			9	3	12		
Total	16	15	31	3.29	0.15	18	13	31	0.31	0.15
Number of dogs above median (2)										
Yes	6	7	13							
No	10	8	18							
Total	16	15	31	0.72	0.72					

^1^ Some respondents replied with “I’m not sure” which was excluded, leading to the variation in totals.

^2^ indicates association was considered significant

^3^ N/A = odds ratio was calculated as infinity

## Discussion

This survey collected information about dog management–including dog movements, vaccination rates, reporting systems and owners’ knowledge and attitudes–in the remote, indigenous communities of the NPA, Australia. Although the survey was limited by small numbers, the information is invaluable for parameterizing a recently developed rabies model, which relies upon variables such as detection time, vaccination rates and dog movement and dog culling compliance, and was previously based on limited empirical data [9]. With these data the model could now reliably inform decision makers on which control strategies would best contain a rabies outbreak in the area and improve Australia’s preparedness against rabies. The information generated is also useful for providing context for dog health and management programs in rural indigenous regions, and could inform the control of other zoonotic diseases.

Study results suggest that men have a significant influence on the health and management of dog populations at the study site, which could be relevant for the mitigation of potential disease spread during and outbreak. The survey was heavily biased towards men because of the survey design and the requirement for informed consent from the person considered to be in charge of the household dog(s) (Table 1). This is likely to be a cultural construct because women may not identify as dog owners, despite living in houses with dogs and potentially being the primary caregiver. The greater proportion of male ownership found in this study was expected: similar studies conducted in rural areas of Tanzania and Taiwan also found that dog ownership was skewed towards men [19, 20]. Men were 9.8 times more likely than women to allow their dogs to roam and it was only men who moved their dogs outside the community. Both of these behaviours could facilitate rabies spread throughout the communities in this region, should an incursion occur. Men were found to own most of the dogs and to allow all of the reported dog movements; therefore they should be targeted for any potential movement bans during a rabies or other contagious disease outbreak, as well as broader educational programs about dog health and disease management.

Despite having limited significant associations due to the small sample size, the association analysis provides valuable insights into certain trends among the dog owning population towards rabies control strategies which could be further investigated in future studies. For example, it is important to know that men are more involved in dog movements between communities compared to women, so as to better target information campaigns and educational programs.

Understanding the current vaccination level in the study dog population provides insight into how feasible a mass vaccination program would be. We found evidence of high willingness and acceptance of a vaccination campaign; all respondents said they would vaccinate their dog(s) during an outbreak. However, the opportunity for vaccination in this remote area of Australia is poor. Only four respondents–who together owned a total of nine dogs–had their dogs vaccinated in the last 12 months (Table 4).

This indicates a current overall vaccination rate of just 12%. The majority of vaccinations coincided with the most recent, local council organized veterinarian visit, which was in 2012 (Table 4). The closest veterinary clinic is located on Thursday Island approximately 35km away in the Torres Strait, but a visit would require a government-issued permit to transport live animals back to mainland Australia. The two closest veterinary clinics on the Australian mainland are located in Weipa and Cairns, a distance (via unsealed roads and river crossings) of approximately 430km and 960km, respectively. As these locations are largely inaccessible to most community members, the majority of dog owners report their sick dog to the AMW despite the limited treatments and advice available. This highlights the lack of available veterinary services in the area and explains the low vaccination rate reported.

Studies conducted in rabies endemic Tanzania revealed similar findings of dog owners having a high willingness to vaccinate their dogs, but having minimal opportunity to do so due to the lack of accessible veterinary facilities [21,22]. This causes suboptimal vaccination coverage and consequently makes rabies eradication problematic [21]. If a rabies incursion occurred in NPA–which has been assessed as a high risk for such an event–accessibility under the current veterinary infrastructure and care services may be the limiting factor for vaccination strategy success rather than dog-owner attitudes towards the strategy. Also, because there is limited dog population control (only about 18% of dogs in this survey were reported to have been de-sexed, Table 2), there is likely to be high a population turnover rate. Developing an effective vaccination response strategy is therefore a priority.

Restricting dog movements, either within or between communities, has previously been used as a strategy to control rabies outbreaks with varying success [4, 9]. To understand how such a strategy would be implemented in the study area, it was vital to understand dog owners’ attitudes towards a potential restriction. Most (90%) of the respondents stated that they would voluntarily impose restrictions on their dogs during an outbreak (Table 3). The 10% of owners that responded in the negative would consist of both those that refuse to control the roaming behaviour of their dogs, and those stating that their dogs were capable of escaping imposed confinement. This highlights the need for more restrictive confinement of dogs during a disease outbreak, with which only around 80% of dog owners in the area would comply. With this compliance being less than 100%, a movement ban may only be effective in slowing the spread–rather than reducing the size–of an outbreak [9]. Despite being potentially ineffective as a standalone strategy, restricting dog movements (including human mediated movements) could still be beneficial in the study area because such restrictions complement vaccination strategies by reducing the chance of rabies spreading to and unvaccinated areas [15]. A previous study conducted in the NPA calculated home ranges of most of the community dogs to be around 0.2–0.4 ha, mainly around the owner’s house [17]. However, some dogs’ home ranges were upwards of 104 ha, spanning multiple communities [17]. These dogs are of particular interest for rabies transmission and need to be targeted for movement bans. This is of particular interest for male dogs because they often occupy larger home ranges than female dogs [17]. Pig hunting represents an interesting problem for rabies transmission because owners and their dogs often assemble from different communities of the NPA, creating conditions for inter-community rabies transmission. As most human mediated movements are for pig hunting, this type of movement should also be targeted during a rabies outbreak.

Mass culling programs have been used in past rabies outbreaks as a strategy to contain and limit the spread of the disease [4, 6, 9, 11]. However, they have been shown to be ineffective. This would likewise be the result in our study area. Although the majority of respondents stated that they would consider euthanizing their dog during an outbreak voluntarily or if suggested by the local AMW, most would only comply if their dog was sick. This immediately decreases the effective population of dog owners willing to allow a cull program. Also, 6% fewer respondents were willing to euthanize their dogs if a non-local official told them to, which suggests resistance to such a strategy and might indicate that some respondents could try to avoid a compulsory cull. Hiding dogs to avoid culling has not only been shown to hinder the effectiveness of such strategies, but also exacerbates disease spread because community members may move latently infected animals into rabies free zones [4, 15]. The number of respondents that stated they would euthanize their dog might also be an overestimate, since the question was hypothetical. Attempts to euthanize dogs for a variety of health and welfare issues in Indigenous communities are usually met with resistance from dog owners. Based on experience from Bali (where dogs were euthanized to limit rabies spread [4, 6, 22]), preliminary model predictions [9] and community attitudes in NPA, euthanasia should only play a minor role in response to a rabies incursion.

The final aim of the study was to estimate a potential detection time for the first rabid dog in the study region. Owners were asked what signs they recognised as indicators of a sick dog. In this study, signs considered typical of rabies (increased aggression, paralysis, hypersalivation, change of voice) did not make up the most distinguishable signs of a sick dog. However, the two categories they fell under (behavioural changes and physical ailments) together accounted for just under half the signs noted as indicators of sick dogs (Table 5). Therefore, rabid dogs are likely to be recognized as ill, since they show obvious signs. More than half the respondents said they would report their sick dog either immediately or within a week. However, of the owners that had sick dogs within the last 12 months, only half of them reported it (Table 5). The owner’s perception of the severity of the illness may influence whether they seek advice and may only do so when they think their dog is very ill. Assuming that the signs of rabies are severe and will finally lead to death, owners are more likely to report the dog and therefore a detection time of less than a week might be a reasonable estimate. This is a more optimistic detection time than has been used in preliminary modelling of the spread of rabies in this region (i.e. 2–4 weeks) [9]. In some other rabies outbreaks, detection times have ranged from 1 to 7 months [23].

Another way to conduct surveillance for rabies is monitoring the number of dog–to–human or dog–to–dog bites, which highlights the distribution and incidence of rabies [23–26]. This type of surveillance could be beneficial as a detection of rabies if dog bites increase. Of the 19 (61%) respondents that said their dog had been bitten by another dog, only two reported the incidents (Table 5). These incidents were reported to the AMW. Therefore, we estimate under-reporting to authorities of dog–to–dog bites of nearly 10-fold. The lack of reporting may be because injuries sustained by the dog bites are not considered abnormal or severe enough by the owners to report, or perhaps due to fear of dog impoudment. Very few dog–to–human bites were recorded in this survey: only two bites, of which one was reported to the AMW. Dog bite surveillance for rabies in the NPA could be a useful tool for detecting rabies in the area, which has been suggested for other rabies-free places such as Lombok, Indonesia [25]. However, the underreporting of dog bites in the NPA could hamper the surveillance for a rabies outbreak as the potential increased aggression in rabid dogs may go unnoticed. Educational programs about rabies, or dog health management in general, could be useful to encourage community members to report any dog–to–dog or dog–to–human bites. This could in turn enhance detection of rabid dogs and improve wound treatment and prevention of human rabies cases, as proven in the Philippines [27]. This is due to the increased awareness provided by such programs, which in turn reduces the amount of victims that are affected by rabies and increases surveillance for the disease [27]. Educational programs have also been successful in Australian Indigenous communities where the increased awareness was directly responsible for improved dog health [28].

The questionnaires could only be completed during working hours because of the need to be accompanied by an indigenous AMW. This limited the amount of dog–owners approached to participate in the survey, resulting in the small number of respondents. Although we achieved a substantial sampling fraction of 11%, the limited sample size meant that the confidence intervals around our estimates were wide. A sample size of 31 would only be sufficient to achieve a margin of error of 14% with 90% confidence, for a 50% proportion. That is, if some proportion is estimated to be 50%, then we can be 90% confident that the population proportion is between 36% and 64%. However, the confidence interval will be narrower for proportions less than or more than 50%; hence the study would be able to achieve a margin of error of 10% for a 15% proportion. Therefore, interpretation of our results should take these uncertainties into account.

A second limitation was that many questions were hypothetical and asked respondents to predict future behaviours. Whilst this gives valuable insights for modelling, such responses need to be considered with caution because actual actions do not always match planned behaviour. Also, the survey does not reflect the selected indigenous communities as a whole and cannot be used to extrapolate information about the wider community’s perceptions. Further investigation needs to be conducted to analyse the long-term impacts of control strategies on the wider community.

Finally, this study did not consider the potential contact between domestic dogs and the wild canine population (dingoes and their hybrids) because it was out of the scope of this study. Further investigation into the interface between domestic and wild dogs is necessary for a more complete preparedness plan against a rabies incursion as these canines could have significant impacts on the size and spread of an outbreak.

### Conclusion

This study successfully collected information on dog health management in remote, northern Australian indigenous communities to better parameterize a rabies epidemiological model. It revealed potential flaws in a dog movement ban, as the compliance of dog owners was not 100%, and emphasised significant shortfalls in veterinary care that would need to be vastly improved during an outbreak to reach the 70% coverage recommended to control rabies. The detection time was optimistic compared to the current model estimation and other rabies detection times seen in previous foreign outbreaks. The study also provided useful information on how the control programs of dog vaccination, culling and movement bans would be accepted by the dog-owning community and highlighted issues to be targeted by educational programs and potential barriers to implementation (such as potential decreased compliance when non-local government officials are involved). Both types of information could be used to better inform decision makers on best practice for containing a potential rabies outbreak in this high-risk region and therefore improve preparedness against a rabies incursion. However, more detailed information is needed to understand potential barriers to implementation of control strategies, and the impacts of rabies control strategies on the wider community.

## Supporting Information

S1 Dataset Data collected in a questionnaire survey of dog owners conducted in the Northern Peninsula Area (NPA), Australia in 2015. (XLSX)

S1 Questionnaire Questionnaire used to survey dog owners in the Northern Peninsula Area (NPA), Australia in 2015. (DOCX)

## References

[1] Hampson K, Coudeville L, Lembo T, Sambo M, Kieffer A, Attlan M, et al Estimating the global burden of endemic canine rabies. PLoS Neglected Trop D. 2015;9(4):e0003709 10.1371/journal.pntd.0003709 PMC4400070 25881058

[2] Knobel DL, Cleaveland S, Coleman PG, Fèvre EM, Meltzer MI, Miranda MEG, et al Re-Evaluating the burden of rabies in Africa and Asia. B World Health Organ. 2005;83(5):360–8. PMC2626230. PMC2626230 15976877

[3] World Health Organisation WHO Expert Consultation on Rabies: Second Report. WHO Technical Report Series. 2013;(982):1–139, back cover. 1444584597; 24069724. 24069724

[4] Putra AA, Hampson K, Girardi J, Hiby E, Knobel D, Mardiana IW, et al Response to a Rabies epidemic, Bali, Indonesia, 2008–2011. Emerg Infect Dis. 2013;19(4):648–51. 10.3201/eid1904.120380 23632033 PMC3647408

[5] Tenzin Ward MP. Review of rabies epidemiology and control in South, South East and East Asia: Past, Present and Prospects for Elimination. Zoonoses and Public Hlth. 2012;59(7):451–67. . 23180493 10.1111/j.1863-2378.2012.01489.x

[6] Windiyaningsih C, Wilde H, Meslin FX, Suroso T, Widarso HS. The rabies epidemic on Flores Island, Indonesia (1998–2003). J Med Assoc Thai. 2004;87(11):1389–93. Epub 2005/04/14. . 15825719

[7] Sparkes J, Fleming PJ, Ballard G, Scott-Orr H, Durr S, Ward MP. Canine rabies in Australia: A review of preparedness and research needs. Zoonoses and Public Hlth. 2014;63:237–53. 10.1111/zph.12142 . 24934203

[8] Clifton M. How not to fight a rabies epidemic: A history in Bali. Asian Biomed. 2010;4(4):663–71.

[9] Dürr S, Ward MP. Development of a novel rabies simulation model for application in a non-endemic environment. PLoS Neglected Trop D. 2015;9(6):e0003876 10.1371/journal.pntd.0003876 PMC4482682. PMC4482682 26114762

[10] Hampson K, Dushoff J, Cleaveland S, Haydon DT, Kaare M, Packer C, et al Transmission dynamics and prospects for the elimination of canine rabies. PLoS Biol. 2009;7(3):e53 10.1371/journal.pbio.1000053 19278295 PMC2653555

[11] Cleaveland S, Beyer H, Hampson K, Haydon D, Lankester F, Lembo T, et al The changing landscape of rabies epidemiology and control. The Onderstepoort Journal of Veterinary Research. 2014;81(2):1–8. 1546008804; . 25005807 10.4102/ojvr.v81i2.731 PMC7612516

[12] Kitala PM, McDermott JJ, Coleman PG, Dye C. Comparison of vaccination strategies for the control of dog rabies in Machakos District, Kenya. Epidemiol and Infect. 2002;129(1):215–22. PMC2869868. 12211590 10.1017/s0950268802006957 PMC2869868

[13] Russell CA, Real LA, Smith DL. Spatial control of rabies on heterogeneous landscapes. PLoS ONE. 2006;1(1):e27 10.1371/journal.pone.0000027 PMC1762310. 17183654 PMC1762310

[14] Smith DL, Lucey B, Waller LA, Childs JE, Real LA. Predicting the spatial dynamics of rabies epidemics on heterogeneous landscapes. P Natl Acad Sci USA. 2002;99(6):3668–72. 10.1073/pnas.042400799 PMC122581. PMC122581 11904426

[15] Townsend SE, Sumantra IP, Pudjiatmoko, Bagus GN, Brum E, Cleaveland S, et al Designing programs for eliminating canine rabies from islands: Bali, Indonesia as a case study. PLoS Neglected Trop D. 2013;7(8):e2372. 10.1371/journal.pntd.0002372 PMC3749988 23991233

[16] ABS. Australian Bureau of Statistics: 2011 Census Data. Australian Bureau of Statistics http://wwwabsgovau/websitedbs/censushomensf/home/Census?opendocument-from-banner=GT Accessed: 2/7/2015. 2011.

[17] Dürr S, Ward MP. Roaming behaviour and home range estimation of domestic dogs in Aboriginal and Torres Strait Islander communities in Northern Australia using four different methods. Prev Vet Med. 2014;117(2):340–57. 10.1016/j.prevetmed.2014.07.008 . 25096735

[18] R Core Team. R: A language and environment for statistical computing. R foundation for statistical computing Vienna, Austria 2014 URL: http://www.R-project.org/

[19] Hsu Y, Severinghaus LL, Serpell JA. Dog keeping in Taiwan: Its contribution to the problem of free-roaming dogs. J Appl Anim Welf Sci. 2003;6(1):1–23. Epub 2003/06/11. 10.1207/s15327604jaws0601_01 . 12795855

[20] Knobel DL, Laurenson MK, Kazwala RR, Boden LA, Cleaveland S. A cross-sectional study of factors associated with dog ownership in Tanzania. BMC Vet Res. 2008;4(1):5- 10.1186/1746-6148-4-5 18230137 PMC2262882

[21] Sambo M, Lembo T, Cleaveland S, Ferguson HM, Sikana L, Simon C, et al Knowledge, attitudes and practices (KAP) about rabies prevention and control: A community survey in Tanzania. PLoS Neglected Trop D. 2014;8(12):e3310 10.1371/journal.pntd.0003310c PMC4256472. PMC4256472 25473834

[22] Bardosh K, Sambo M, Sikana L, Hampson K, Welburn SC. Eliminating rabies in Tanzania? Local understandings and responses to mass dog vaccination in Kilombero and Ulanga Districts. PLoS Neglected Trop D. 2014;8(6):e2935 10.1371/journal.pntd.0002935 PMC4063706. PMC4063706 24945697

[23] Townsend SE, Lembo T, Cleaveland S, Meslin FX, Miranda ME, Putra AAG, et al Surveillance guidelines for disease elimination: A case study of canine rabies. Comp Immunol Microb. 2013;36(3):249–61. 10.1016/j.cimid.2012.10.008 PMC3693035 23260376

[24] Frey J, Mindekem R, Kessely H, Doumagoum Moto D, Naïssengar S, Zinsstag J, et al Survey of animal bite injuries and their management for an estimate of human rabies deaths in N’djaména, Chad. Trop Med Int Health. 2013;18(12):1555–62. 10.1111/tmi.12202 24118491

[25] Mustiana A, Toribio J-A, Abdurrahman M, Suadnya IW, Hernandez-Jover M, Putra AAG, et al Owned and unowned dog population estimation, dog management and dog bites to inform rabies prevention and response on Lombok Island, Indonesia. PLos ONE 2015;10(5): e0124092 10.1371/journal.pone.0124092 25932916 PMC4416720

[26] Tenzin, Dhand NK, Gyeltshen T, Firestone S, Zangmo C, Dema C, et al Dog bites in humans and estimating human rabies mortality in rabies endemic areas of Bhutan. PLoS Neglected Trop D. 2011;5(11):e1391 10.1371/journal.pntd.0001391 PMC3222627. PMC3222627 22132247

[27] Lapiz SMD, Miranda MEG, Garcia RG, et al Implementation of an intersectoral program to eliminate human and canine rabies: The Bohol Rabies Prevention and Elimination Project. Zinsstag J, ed. PLoS Neglected Trop D. 2012;6(12):e1891 10.1371/journal.pntd.0001891 PMC3516573 23236525

[28] Constable S, Dixon R, Dixon R. Learning Preferences and Impacts of Education Programs in Dog Health Programs in Five Rural and Remote Australian Indigenous Communities. Aust J Indigenous Educ. 2011;40:48–58. 10.1375/ajie.40.48

