# Supplementary material for: A Survey of Dog Owners in Remote Northern Australian Indigenous Communities to Inform Rabies Incursion Planning
Source: PLoS Negl Trop Dis. 2016 Apr 26;10(4):e0004649. doi: 10.1371/journal.pntd.0004649 (PMC4846002; doi:10.1371/journal.pntd.0004649)
Supplement: S1 Questionnaire — (DOCX) [file pntd.0004649.s002.docx]

**1. Demographics**

1a) Are you a dog owner? Yes / No

Gender: M/F

(Don’t ask just circle)

1b) Human demographics

| Age | |  |
| --- | --- | --- |
| 18-29 |  | |
| 30-39 |  | |
| 40-49 |  | |
| 50-59 |  | |
| 60-69 |  | |
| 70 and above |  | |

| Employment | |
| --- | --- |
| Casual |  |
| Part-time |  |
| Full-time |  |
| Unemployed |  |

| Ethnicity | |
| --- | --- |
| Aboriginal |  |
| Torres Strait Islander |  |
| Non-indigenous |  |
| Other |  |

| Highest Education | |
| --- | --- |
| Yr 10 |  |
| Yr 12 |  |
| Apprentice |  |
| TAFE |  |
| University |  |
| Other |  |

1c) Where do you get most of your information regarding animal or human health from?

Animal

Management Worker

Newspaper

Friends/Family

Internet

Home TV

Radio

Vet

Other ____________________________________________

1d) Dog demographics currently - owned or in household:

| Dog Name/ID | Previously Collared | Male or female? | De-sexed? | Age | Where was this dog born |
| --- | --- | --- | --- | --- | --- |
| 1 |  |  |  |  |  |
| 2 |  |  |  |  |  |
| 3 |  |  |  |  |  |
| 4 |  |  |  |  |  |
| 5 |  |  |  |  |  |
| 6 |  |  |  |  |  |

**2. Detection:**

ANY DOG

2a) How would you describe a sick dog? __________________________________________________

_________________________________________________________________________________

2b) Which of these dogs do you think are sick (pictures)?

E

A

B

C

D

(may have more options depending on how many photos)

None

2c) Who would you report a sick dog to?

Animal Management Worker

Vet

No one

Other ___________

Police

Council

Ranger

State Government

Federal Government

YOUR OWN DOG

2d) When was the last time you took any of your dogs to the vet? (now focus on the most recent dog that went to the vet)

Yes

No

Last week

Last month

Last year

More than a

year ago

Never

Don’t know

2e) Has your dog been sick in the last 12 months?

2f) If so what signs did you see? ________________________________________________________

_________________________________________________________________________________

2g) Who did you report it to?

Animal Management Worker

Vet

No one

Other ___________

Police

Council

Ranger

State Government

Federal Government

2h) Did they receive any treatment?

No

Needle

Tablet

Cream

Other __________

I don’t know

Yes, but I don’t know what

2i) What was the outcome?

Got better in a few days

Got better in a few weeks

Died naturally

Got put down/euthanized

2j) If your dog was sick or if you saw a sick dog how long would you wait until you reported it/ How long have you waited?

Immediately

The next day

Within a week

The next week

More than 2 weeks

2k) Has this dog ever had a needle?

Yes

No

2l) What was it for?­­­­­­­___________________________________________

2m) Where did it get the needle (geographical location)?

Cairns

Other ___________________

Thursday Island

Seisia

New Mapoon

Bamaga

Injinoo

Umagico

2n) How long ago?

________________________________

Yes

No

2o) Does a Vet visit regularly? ­­­

Yes

No

2p) Is it advertised that a vet is coming?

2q) If you needed vet services would you visit:

Other ______________________

None

Thursday Island

Cairns

Weipa

2r) Have you been bitten by a dog in the last year?

Yes

No

2s) Who did you report it to?

Council

Animal Management Worker

Police

Federal Government

State Government

Doctor

Ranger

No one

Other ___________

2t) How long did you wait until you reported it?

Immediately

The next day

Within a week

The next week

More than 2 weeks

Yes

No

2u)Has your dog(s) been bitten by another dog in the last year?

2v) Who did you report it to?

Animal Management Worker

Vet

No one

Other ___________

Police

Council

Ranger

State Government

Federal Government

2w) How long did you wait before you reported it?

Immediately

The next day

Within a week

The next week

More than 2 weeks

**3. Movement Control**

3a) Do you allow your dog to roam?

Yes

No

Within the community

To neighbouring communities

Into the bush

3b) Where does your dog roam?

3c) How often does your dog come home?

Daily

Every second day

Weekly

Monthly

Over a month

3d) Have you taken your dog outside your community in the last 12 months?

Yes

No

3e) What for? ________________________________

Within NPA

Outside NPA: where__________________

3f) Where did you take them?

3g) How long did you stay there with the dog?

Back within the day

Overnight

A few

days

A week

A month

Over a month

Permanently

3h) Did you bring the dog back home?

Yes

No

3i) Do you ever:

Keep your dog inside

Chain your dog up

Close your gate

None of the above

3j) If there was a disease outbreak would you voluntarily:

None of the above

Keep your dog inside

Chain your dog up

Close your gate

3k) Would you restrict your dog if you were told to by council/government?

Yes

No

3l) If there was a severe disease outbreak would you voluntarily allow your dog to be euthanized?

Yes

I don’t know

No

3m) Would you put your dog to sleep if you were told to by council/government?

I don’t know

No

Yes

3n) If there was a disease outbreak would you voluntarily vaccinate your dogs?

I don’t know

No

Yes

3o) If there was a disease outbreak would you vaccinate your dogs if you were told to by the council/government?

I don’t know

No

Yes
